# Antiviral potency and functional analysis of tetherin orthologues encoded by horse and donkey

**DOI:** 10.1186/1743-422X-11-151

**Published:** 2014-08-27

**Authors:** Xin Yin, Miaomiao Guo, Qinyong Gu, Xingliang Wu, Ping Wei, Xiaojun Wang

**Affiliations:** College of Veterinary Medicine, Northeast Agricultural University, Harbin, China; State Key Laboratory of Veterinary Biotechnology, Harbin Veterinary Research Institute, the Chinese Academy of Agriculture Sciences, Harbin, China

**Keywords:** Equine tetherin, Antiviral activity, EIAV, NF-κB

## Abstract

**Background:**

Tetherin is an interferon-inducible host cell factor that blocks the viral particle release of the enveloped viruses. Most knowledge regarding the interaction between tetherin and viruses has been obtained using the primate lentiviral system. However, much less is known about the functional roles of tetherin on other lentiviruses. Equine infectious anemia virus (EIAV) is an important macrophage-tropic lentivirus that has been widely used as a practical model for investigating the evolution of the host-virus relationship. The host range of EIAV is reported to include all members of the *Equidae* family. However, EIAV has different clinical responses in horse and donkey. It’s intriguing to investigate the similarities and differences between the tetherin orthologues encoded by horse and donkey.

**Results:**

We report here that there are two equine tetherin orthologues. Compared to horse tetherin, there are three valine amino acid deletions within the transmembrane domain and three distinct mutations within the ectodomain of donkey tetherin. However, the antiviral activity of donkey tetherin was not affected by amino acid deletion or substitution. In addition, both tetherin orthologues encoded by horse and donkey are similarly sensitive to EIAV Env protein, and equally activate NF-κB signaling.

**Conclusion:**

Our data suggest that both tetherin orthologues encoded by horse and donkey showed similar antiviral activities and abilities to induce NF-κB signaling. In addition, the phenomenon about the differential responses of horses and donkeys to infection with EIAV was not related with the differences in the structure of the corresponding tetherin orthologues.

## Background

Tetherin, an IFN-inducible, glycosylated restriction factor and type II membrane protein, is responsible for inhibiting the release of human immunodeficiency virus (HIV) and other enveloped viruses from infected cells [1, 2]. This viral release restriction factor contains a cytoplasmic N-terminal region, a transmembrane region, a coiled-coil ectodomain and a C-terminal glycosylphosphatidylinositol (GPI) anchor. Among the known proteins, this double-anchored topology is relatively unique and important for its antiviral restriction activity [3]. Tetherin incorporates one of its two membrane anchors into viral membranes, and thereby traps enveloped viral particles on the surface of infected cells, leading to their internalization and degradation. As another function, it was recently reported that human tetherin acts as an innate sensor to activate NF-κB and promote pro-inflammatory gene expression [4, 5].

In turn, enveloped viruses have evolved different mechanisms to antagonize this restriction by tetherin. Influenza virus disrupts the interferon pathway to reduce the production of tetherin, thereby antagonize the antiviral activity of tetherin [6]. Moreover, several different virus-encoded proteins have been implicated to counteract tetherin: human immunodeficiency virus type 1 (HIV-1) Vpu, the envelope glycoproteins encoded by HIV-2, SIVtan, feline immunodeficiency virus (FIV), EIAV, Ebola virus (GP), herpes simplex virus 1 (HSV-1) glycoprotein M, simian immunodeficiency virus (SIV) Nef, and Kaposi’s sarcoma-associated herpes-virus (KSHV) K5 [7–16]. Recently it was shown that herpes simplex virus type 1 (HSV-1) induces tetherin mRNA degradation via its virion host shutoff activity to counteract tetherin restriction [17].

Equine infectious anemia virus (EIAV), which belongs to the *Retroviridae* family, is a non-primate enveloped virus that has been reported to infect all members of the *Equidae* family [18, 19]. The clinical cases and virus evolution have been well documented in horses, ponies, donkey and mules. However, susceptible to infection, donkeys do not develop clinical response. In addition, lower amounts of plasma associated virus are detected in donkeys compared to horses infected with EIAV [20]. Recently, we have cloned the tetherin homologue of horse, and reported that horse tetherin can restrict EIAV release from infected cells and that its antiviral activity is antagonized by EIAV Env [7]. Thus, it is intriguing to investigate the similarities and differences between the tetherin orthologues encoded by horse and donkey.

In this study, we investigated the similarities and differences between both equine tetherin orthologues. Donkey tetherin has a shorter sequence compared to those of its homologues. The amino acid sequence of donkey tetherin differs from that of horse tetherin in the transmembrane domains and ectodomains. However, both of them displayed similar antiviral activity against EIAV and HIV-1. In addition, the distinct amino acids between two equine tetherin orthologues didn’t govern the sensitivity to antagonism by EIAV Env. Interestingly, both equine tetherin orthologues lacking the tyrosine motif within cytoplasmic tail could activate the NF-κB signaling.

## Results and discussion

### The tetherin homolog encoded by *donkey (Equus asinus)*is different from that of horse *(Equus caballus)*

**Figure 1:**
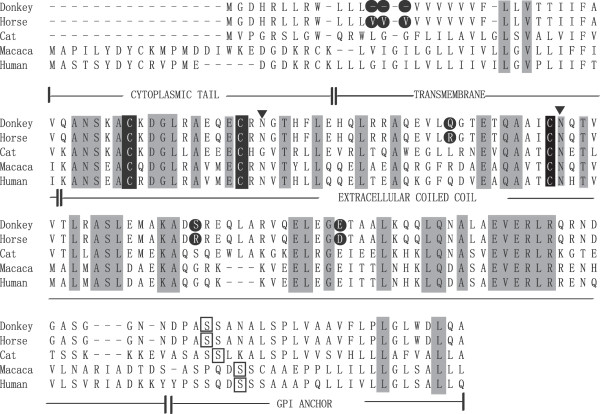
**Deduced amino acid sequences of equine tetherin isofroms.** Alignment of the amino acid sequences of donkey, horse (KF899866), cat (AB564550), rhesus macaque (HQ596987), and human (NM_004335) tetherin homologs. Amino acid residues conserved in all the above-described sequences are shaded in light gray. The different amino acids between donkey and equine tetherin are shown in a black circle. Three Cys residues which are important for dimerization, are shown in a black background. Two putative N-glycosylation sites are marked with a black triangle.

Due to their antiviral activity and immunological properties, human tetherin and its related homologues have become attractive research targets [21, 22]. Recently, we have cloned and characterized the tetherin homolog of horse [7]. To evaluate species-specific differences of tetherin in different *equid* species, we isolated total RNA from donkey and horse macrophages and amplified the complete coding regions of donkey tetherin. An approximately 480-bp product was amplified by RT-PCR. Sequence analysis of the amplification products demonstrated that the entire amino acid sequences of donkey and horse tetherin aligned, except for a deletion (indel) of three valine residues at positions 13–15 in donkey tetherin. The aligned amino acids varied at the following three positions: 65, 92 and 105 (Figure 1). Interestingly, the transmembrane regions of donkey and equine tetherin contain an unusually high percentage of valine residues. Valine is an aliphatic and extremely hydrophobic amino acid, and hydrophobic amino acids are important in the binding/recognition of hydrophobic ligands, such as lipids [23]. Thus, one possibility for the valine-rich nature of equine tetherin orthologues may be related to its dependence on valine-mediated protein-protein and protein-lipid interactions. It has been reported that the donkey (*Equus asinus*) was one of the first domesticated members of the *Equidae* family, while its common ancestor may have diverged from the horse (*Equus caballus*) approximately 2 million years ago [24]. Thus, our results demonstrated that two equine tetherin orthologues have evolved independently after speciation within the *Equidae* family.

### Analysis of the post-translational modification and subcellular localization of two equine tetherin orthologues

**Figure 2:**
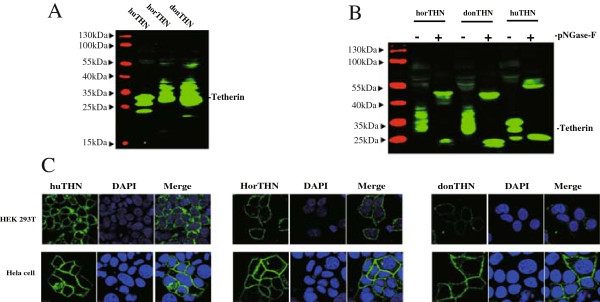
**Post-translational modification and subcellular localization of two equine tetherin orthologues. (A)** The expression plasmids for human, horse or donkey tetherin homologues (1 μg) were transfected into HEK 293 T cells. Intracellular expression of tetherin was analyzed by Western blotting using anti-HA antibody. **(B)** 293 T cells were transfected with the expression plasmids for donkey, horse or human tetherin. The cell lysates were treated with (+) or without (−) PNGase Ffor de-N-glycosylated reaction, and then detected with anti-HA antibody by Western blotting. **(C)** Three tetherin homologues expressed in HEK293T and Hela cells were stained with an anti-HA antibody (green). The cell nuclei were stained with DAPI stain (blue).

It has been previously reported that human tetherin is modified by N-linked glycosylation at two sites [25]. Therefore, human tetherin could be detected as three bands that corresponded to double, single and non-glycosylated forms, from top to bottom [22, 25, 26]. However, compared with human tetherin, the two equine tetherin orthologues migrated as multiple glycoforms in our western blot analysis. Furthermore, we analyzed the glycosylation of equine tetherins by using PNGase F. As shown in Figure 2B, PNGase F treatments reduced the apparent molecular weights of all tetherins, resulting in deglycosylated proteins. It should be noted that an additional protein band was present above the deglycosylated proteins, which closely matched the predicted molecular weight of a tetherin dimer. The result demonstrated that human and equine tetherins may have distinct glycosylation profiles, both equine tetherin orthologues are heavily modified post-translationally by N-linked glycosylation.

The plasma membrane localization of human tetherin and its colocalization with virus particles are vital to its restriction function, as it need tether newly formed virions to the cell membrane [27]. Due to the truncated transmembrane domain in donkey tetherin, it is not clear whether the subcellular localization of donkey tetherin is different from that of horse tetherin. To this end, HEK293T and HeLa cells were transfected with human, horse and donkey tetherins respectively, and the subcellular localizations of these constructs were analyzed by confocal immunofluorescence. As shown in Figure 2C, three tetherin proteins were preferentially localized at the plasma membrane. Three valine deletions in the transmembrane domain of donkey tetherin had no effect on its subcellular localization. Our results suggested that the plasma membrane localization was an inherent characteristic of two equine tetherin orthologues. This phenomenon is consistent with the model suggesting that tetherin restricts virus release by directly retaining the viral particle at the plasma membrane [28].

### EIAV viral particle release is blocked by the two equine tetherin orthologues

**Figure 3:**
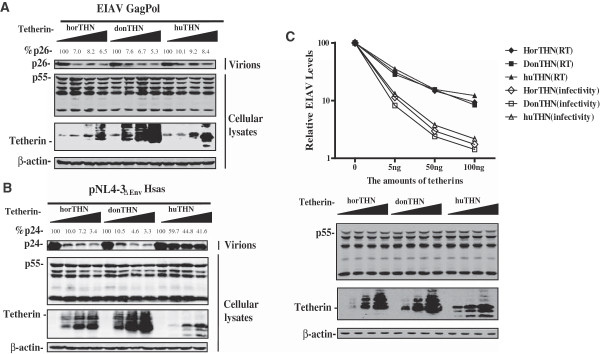
**The antiviral acitivity of equine tetherin orthologues.** EIAV GagPol expressing construct **(A)** or pNL-r-HSAS **(B)** was transfected into 293 T cells along with increasing amounts of huTHN,horTHN, or donTHN (0, 10, 100, 200 ng). Cells were dissolved at 48 h after transfection and then subjected to SDS-PAGE. Viral proteins were analyzed by Western blotting using anti-EIAV serum, or a HIV p24^Gag^ monoclonal antibody (no. 3537). Intracellular expression of tetherin was detected by Western blotting using anti-HA antibody. **(C)** The EIAV reporter provirus pONY8.1luc, pGagPol_UK3_, and VSV-G were co-transfected with increasing amount of human, horse or donkey tetherins into HEK293T cells. Expression of viral Gag protein and tetherins in cells were assessed by Western blotting. Amounts of EIAV particles in supernatants were determined by measuring viral reverse transcriptase (RT) activity. Levels of infectious HIV-1 were assessed by infecting the HEK293T cells. Values that were obtained from experiments with EIAV reporter constructs alone are set as 100.

EIAV has different clinical responses in different *equid* species [20]. It is intriguing to investigate whether or not the differences between the clinical manifestations of EIAV in horse and donkey may be attributed to the distinct antiviral activity of the corresponding tetherin orthologues. To directly compare the restriction activity of the two equine tetherin orthologues against the release of retroviral particles (EIAV, HIV-1), HEK293T cells were transfected with constructs to produce these virus-like particles (VLPs) along with increasing amounts of plasmid for the expression of equine or human tetherins. The amount of viral particles released into the culture supernatant was analyzed by Western blotting. As shown in Figure 3A and B, the yield of intracellular viral GagPol proteins was unaffected by the presence of tetherins in the HEK293T cells. Human tetherin potently inhibited the budding of EIAV VLPs, except for HIV-1 particles expressing Vpu. Meanwhile, the release of these virus-like particles was inhibited by both equine tetherin orthologues in a dose-dependent manner. These results indicated that both equine tetherin orthologues showed similar antiviral activities against both HIV and EIAV. These findings further indicate that the structural configuration of the tetherin protein, rather than its primary sequence, is crucial for its antiviral activity.

In addition to restrict the release of viral particles from the cell surface, human tetherin was also reported to impair the infectivity of these progeny virions [29]. To further investigate whether both equine tehterin orthologues displayed distinct effects on the infectivity of cell-free virions. HEK293T cells were co-transfected with constructs to produce VSV-G pseudotyped EIAV virions along with increasing amounts of plasmid for the expression of equine or human tetherins. The amounts of progeny virions in the culture supernatants were determined by measuring viral reverse transcriptase activity; the levels of infectious EIAV particles in the supernatants were determined by infecting the HKE293T cells. As shown in Figure 3C, our study further demonstrated that both horse and donkey tetherins also could diminish the infectivity of the cell-free virus particles, which is consistent with the results reported by other group [29]. It is noted that the infectivity of vrions produced in HEK293T cells expressing donkey tetherin slightly lower than that in HEK293T cells expressing horse tetherin, but no significant difference was determined. Therefore, it is speculated that in addition to reduce the production of cell-free viruses, both horse and donkey tetherins also impair the infectivity of EIAV particles.

### Two equine tetherin orthologues are similarly sensitive to EIAV Env

**Figure 4:**
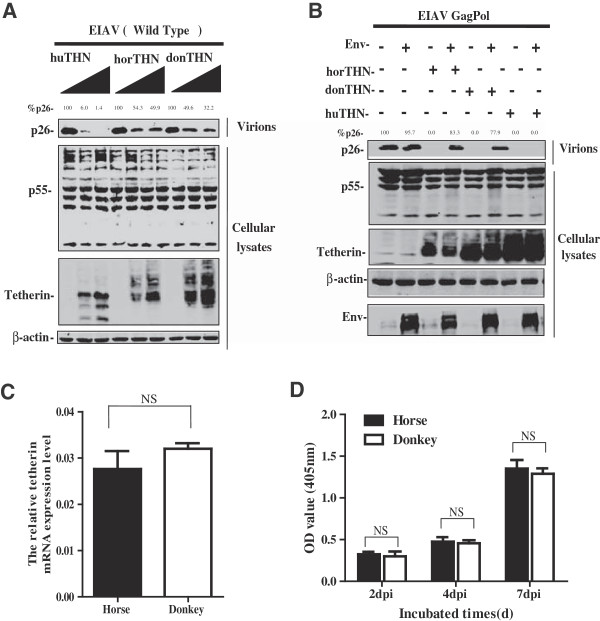
**Two equine tetherin orthologues are similarly sensitive to EIAV Env. (A)** The pCMV-EIAV plasmids were transfected into HEK293T cells along with increasing amounts of the indicated tetherin constructs (0, 50, 100 ng). **(B)** An EIAV GagPol expression construct (2.5 μg) and the tetherin expression vectors as indicated were transfected into HEK 293 T cells in the presence or absence of 1 μg of EIAV Env expression vector. Cells were dissolved at 48 h after transfection and then subjected to SDS-PAGE. Viral proteins were analyzed by Western blotting using anti-EIAV serum. Intracellular expression of tetherin was detected by Western blotting using anti-HA antibody. **(C)** Tetherin and β-actin mRNA expression in horse and donkey macrophage cultures were quantified by real-time PCR. Histograms represent the averages from three independent experiments (n = 3). **(D)** Both horse and donkey monocyte-derived macrophages (2 × 10^6^) were infected with EIAV at 1 × 10^3^ TCID_50_. Reverse transcriptase activity in supernatants from EIAV-infected horse and donkey macrophages was determined by measuring the reverse transcriptase activity using an RT activity kit (Reverse Transcriptase Assay, Colorimetric kit, Roche, Switzerland) as per the manufacturer’s protocol.

Human tetherin can be expressed as two orthologues derived by alternative translation initiation [30, 31]. Importantly, the short tetherin isoform is less sensitive HIV-1Vpu-mediated antagonism [30]. We previously reported that horse tetherin is sensitive to antagonism by EIAV Env proteins. As shown above, six amino acid differences exist between the sequences of the two equine tetherin orthologues. Perhaps the sensitivity of both equine tetherin orthologues to EIAV Env-mediated antagonism is different. This hypothesis was tested by co-transfecting HEK293T cells with plasmids expressing human, donkey or horse tetherin along with a construct expressing the full EIAV genome. The amount of virions that were released into culture supernatant was analyzed by Western blotting. Human tetherin could inhibit wild-type EIAV release from HEK293T cells; however, both donkey and horse tetherin did not efficiently restrict viral particle release from HEK293T cells. A clear dose–response effect of both equine tetherin orthologues on EIAV viral production was not observed (Figure 4A). To further compare the ability of EIAV Env to counteract with the two equine tetherin orthologues, HEK293T cells were transfected an expression plasmid for EIAV Env in combination with the EIAV GagPol construct and an expression plasmid for donkey, horse or human tetherin, and the amount of virions released into the HEK293T cell supernatant was analyzed by Western blotting. As shown in Figure 4B, the exogenous expression of the EIAV Env protein significantly rescued viral particle release from the HEK293T cells transfected with donkey or horse tetherin. Therefore, these results demonstrated that two equine tetherin orthologues are similarly sensitive to EIAV Env and that the activity of EIAV Env protein against donkey tetherin was not affected by these amino acid deletions and substitutions. To further support our results, the endogenous expression levels of tetherin and viral replication capacity in both horse and donkey macrophage cultures were compared. However, there was no significant difference in expressions between two cell types (Figure 4C). In addition, consistent with previous reports, both types of cells supported very similar levels of viral replication (Figure 4D). Overall, this observation suggested that the differential responses of horses and donkeys to infection with EIAV were not related with the primary interaction between EIAV and equine tetherin orthologues.

### Both donkey and horse tetherins activate NF-κB

**Figure 5:**
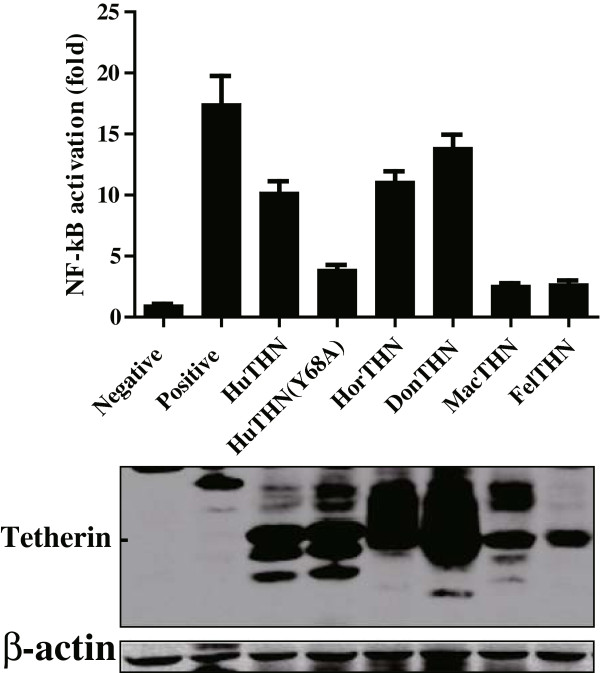
**Both donkey and horse tetherins activate NF-κB.** The indicated constructs were transfected into HEK293T cells with pNF-κB-Luc plasmid and pRL-TK. The transfected cells were lysed and quantified for luciferase activity 36 h post-transfection. Horse MAVS used as a positive control for NF-kB activation.

Human tetherin has been reported to play a role in induction of the pro-inflammatory response regulator NF-κB. However, unlike long tetherin isoform, the truncated tetherin isoform does not activate NF-κB. Specifically, the tyrosine motif within cytoplasmic tail of the long human isoform is found to be critical for NF-κB activation [30]. To explore whether equine tetherin orthologues had differential abilities to activate NF-κB, a luciferase reporter assay was employed in HEK 293 T cells transiently expressing tetherin. Consistent with previous report, over-expression of human tetherin led to NF-kB activation approximately 10–20 folds over control cells transfected with the empty vector alone [4, 30]. However, compared to the wild type human tetherin, the ability of the tyrosine mutant (AxA) to activate NF-κB was reduced. Surprisingly, although equine tetherin orthologues don’t have this dual-tyrosine motif in its cytoplasmic domain, both of them could activate NF-κB signaling to levels similar to those seen with human tetherin (Figure 5). Additionally, macaque and feline tetherins showed negligible ability to induce NF-κB signaling. It’s reported that the interaction between human tetherin and TRAF6 was essential to activation of NF-κB and this interaction was actually determined by the adjacent endocytic motif YXY motif [5]. Therefore, we speculate that equine tetherins might active NF-κB signaling through different signal pathway.

## Conclusions

In conclusion, we showed that the tetherin homolog encoded by donkey (*Equus asinus*) is different from that of horse (*Equus caballus*). However, both equine tetherin orthologues displayed apparent antiviral activity against EIAV and HIV-1. In addition, the sensitivity of both equine tetherin orthologues to EIAV Env-mediated antagonism is similar. Surprising, both equine tetherin orthologues without dual-tyrosine motif could potently activate the NF-κB signaling.

## Materials and methods

### Ethics statement

Donkeys and horses used in this study were approved by the Institutional Animal Care and Use Committee (IACUC) of the Harbin Veterinary Research Institute (HVRI), Chinese Academy of Agricultural Sciences. There were no animals sacrificed specifically for this study. Donkey and horse monocyte-derived macrophages (MDMs) were isolated from 200 mL peripheral blood taken from the jugular vein by veterinarians.

### Cell culture

Human embryonic kidney (HEK) 293 T cells and HeLa cells were maintained at 37°C in a 5% CO_2_ incubator in Dulbecco’s modified Eagle’s medium (HyClone, USA) supplemented with 10% fetal bovine serum and penicillin/streptomycin (100units/ml).

Donkey and horse macrophage cultures derived from peripheral blood mononuclear cells were prepared from EIAV sero-negative animals. The purified donkey and horse primary macrophage cells were maintained in RPMI 1640 supplemented with 10% heat-inactivated horse serum, 30% heat-inactivated fetal bovine serum and penicillin/streptomycin (100 units/ml) (Gibco, USA).

### Plasmids

Human and horse tetherin constructs (pEF-huTHN and pEF-horTHN) were constructed in our laboratory as previously described [7]. The pNL-r-HSAS plasmid was obtained from Drs. Beth Jamieson and Jerome Zack through the NIH AIDS Reagent Program. Plasmid pLG3-8 is a full-length molecular clone of EIAV_FDDV_12 and was constructed from the provirus DNA of EIAV_FDDV12_. pCMV-EIAV is a derivative of the pLG3-8 plasmid. The U3 region of the 5’ LTR was replaced with the cytomegalovirus early promoter amplified from pcDNA3.1 plasmid (Invitrogen) by digestion with the restriction enzymes *Mlu*I and *Xba*I. Using these constructs as templates, EIAV GagPol was amplified from proviral plasmids and inserted as *Eco*RI-*Not*I fragments into the VR-1012 vector (a kind gift from Dr. Xiaofang Yu). pNF-κB-Luc was purchased from Stratagene. Tetherin chimeric constructs were generated from pEF-huTHN and pEF-horTHN by using the overlapping PCR, and then confirmed by nucleotide sequencing.

### Identification and Cloning tetherin homologue from donkey macrophages

The primers (forward primer 5’-ATGGGGGACCACAGGCTGCTGAGAT-3’, reverse primer 5’-TCAGGCCTGCAGATCCCAGAGGCCC-3’) were designed using horse tetherin sequence. The cDNA fragment of potential donkey tetherin was amplified by RT-PCR from total RNA extracted from donkey macrophage cells. The amplified fragments were purified and cloned into the pEF-Flag-HA. The resultant expression plasmid for donkey tetherin was named pEF-donTHN. To determine the initiation site of donkey tetherin, 5’-RACE was performed using a 5’-Full RACE Core Set (Takara) as described previously [7]. The DNA fragments encoding the full-length horse MAVS were amplified by RT-PCR using the primers (forward primer 5’CCAAGAATTCTATGACGGTTGCCGAGGACAAGACTT3’, reverse primer 5’CCAATCTAGAtcaCTGGAGCAGGCGCCTACGGTACAGC3’) and subcloned into EF-Flag-HA, resulting in the Flag/HA-fusion expression constructs pEF-HorMAVS.

### Measurement of tetherin mRNA expression by real-time PCR

Total RNA was extracted from donkey and horse macrophage cells (2 × 10^6^). 100 ng of total RNA was subjected to reverse transcription, and then subjected to real-time PCR using a SYBR Green® Master Mix kit (Invitrogen) according to the manufacturer’s protocols. The procedure to measure of tetherin mRNA expression by Real-time RT-PCR was described previously [7].

### Virion release assays and Western blotting

Virus-like particle (VLP) release assays were performed by transfecting HEK293T cells. Briefly, HEK293T cells were cultured in 6-well plates and cotransfected with 5 μg of pEIAV-GagPol, or pNL-r-HSAS, and different amounts (from 100 ng to 2000 ng) of horse, human or donkey tetherin expression vector or empty vector using standard calcium phosphate transfection. Forty-eight hours post-transfection, the cells were harvested, lysed in lysis buffer (Tris–HCl pH 7.5, 50 mM NaCl, 5 mM EDTA and 1% Triton X-100) and then centrifuged at 10,000 × g for 5 min to remove the cell nuclei. The virus-containing supernatants were harvested and clarified by low-speed centrifugation at 10,000 × g for 10 min at 4°C. The VLPs that were released into the culture medium were further purified by centrifugation (20,000 × g for 2 h at 4°C) and then resuspended in phosphate-buffered saline (PBS). The intracellular Gag and tetherin proteins and pelleted virion particles were analyzed by Western blotting. The human, horse, and donkey tetherin proteins tagged with HA were detected with a mouse monoclonal anti-HA antibody (Sigma). EIAV Gag and Env were detected using EIAV-positive serum. HIV-1 Gag was detected with an anti-P24 monoclonal antibody, and β-actin was detected with an anti-β-actin antibody (Sigma). Alexa Fluor 800-labeled goat anti-mouse IgG and goat anti-horse IgG (Odyssey) were used as secondary antibodies. The blots were imaged with Odyssey using the 800 nm channel to visualize IRDye 800CW. Each experiment was performed independently at least three times.

### Measuring virus infectivity

293 T cells cultured in 6-well plates were transfected with 1.5 ug pGagPol_UK3_, 1.5 ug pONY8.1luc (a kind gift from Dr. Carsten Munk), 0.5 ug the vesicular stomatitis virus G protein (VSV-G) expression plasmid, and increasing amount of human, horse or donkey tetherins by a standard calcium phosphate transfection. Forty-eight hours post-transfection, virus-containing supernatants were harvested and clarified by low-speed centrifugation at 12000 × g for 10 min at 4°C and used to infect naive 293 T cells after quantifing the reverse transcriptase activity of virus by using a RT kit (Roche, Germany). After Forty-eight hours post-infection, the infected 293 T cells were lysed in lysis buffer containing 50 mM Tris–HCl (PH7.4), 150 mM NaCl, 3 mM EDTA, and1%Triton X-100, the cytosolic fraction was used to determine luciferase activity with a luciferase assay kit (Promega).

### Luciferase assay

HEK293T cells cultured in 24-well plates were co-transfected with the indicated tetherin constructs (200 ng) and pNF-κB-Luc plasmid (50 ng) in combination with 10 ng/well pRL-TK (as an internal control to normalize the transfection efficiency, Promega). Luciferase assays were performed at 36 h after transfections. Firefly luciferase and Renilla luciferase activities were quantified using the Dual-Luciferase Assay Kit (Promega) as per manufacturer’s instructions. Statistical analysis was performed using PRISM (GraphPad).
